# Impact of the Family Environment on the Emotional State of Medical Staff During the COVID-19 Outbreak: The Mediating Effect of Self-Efficacy

**DOI:** 10.3389/fpsyg.2020.576515

**Published:** 2020-10-09

**Authors:** Na Hu, Ying Li, Su-Shuang He, Lei-Lei Wang, Yan-Yan Wei, Lu Yin, Jing-Xu Chen

**Affiliations:** ^1^Beijing Huilongguan Hospital, Peking University Huilongguan Clinical Medical School, Beijing, China; ^2^Beijing Children’s Hospital, Capital Medical University, National Center for Children’s Health, Beijing, China

**Keywords:** COVID-19, medical staff, family environment, self-efficacy, anxiety symptoms, depression symptoms

## Abstract

During the outbreak of the coronavirus disease 2019 (COVID-19), the medical staff was facing severe work pressure, which led to a negative emotional state. The purpose of this study was to explore the relationship between the family environment and the emotional state of the medical staff members during the COVID-19 outbreak. Due to the importance of self-efficacy in regulating mental health, the mediating role of self-efficacy in the association between family environment and emotional state was also explored. A cross-sectional survey was performed, using an online questionnaire, on 645 medical staff who participated in the epidemic prevention and control tasks during the COVID-19 outbreak in Beijing. Family environment, self-efficacy, anxiety, and depressive symptoms were measured by the Family Environment Scale-Chinese Version (FES-CV), the General Self-Efficacy Scale (GSES), the Generalized Anxiety Disorder Scale-7 (GAD-7), and the Patient Health Questionnaire-9 (PHQ-9), respectively. Correlation analysis and mediating effect analysis were used to explore the relationships between them. First, a higher prevalence of anxiety (39%) and depressive (33%) symptoms were confirmed among the medical staff. Second, the symptoms of anxiety and depression were negatively correlated with the dimensions of cohesion and expressiveness and positively correlated with the dimensions of conflict in the FES-CV scale. Third, self-efficacy significantly mediated the association between the family environment and anxiety symptoms (*P* < 0.001) as well as the family environment and depressive symptoms (*P* < 0.001). These findings show that a negative family environment was the main predictor of symptoms of anxiety and depression in the medical staff during the COVID-19 outbreak. Furthermore, we found that self-efficacy played a critical mediating role between the family environment and the symptoms of anxiety and depression. Our study also indicates that improvements in the family environment benefit the mental health care of the medical staff, and high self-efficacy enhances this effect.

## Introduction

The outbreak of coronavirus disease 2019 (COVID-19) continues to attract worldwide attention (Wang et al., 2020). To date, COVID-19 cases have been confirmed in more than 200 countries around the world, and it has become a public health emergency of international concern. Many people who have directly faced this large-scale public crisis, especially the medical staff involved in the prevention and control of this epidemic, showed anxiety symptoms (Huang et al., 2020). They faced a high risk of getting infected at their workplace and the possibility of their family members at home getting infected through them (Xiang et al., 2020). Medical staff working in a high-pressure environment suffer from psychological problems, especially anxiety and depression (Kang et al., 2015; Wang et al., 2020). Although guidelines on the care of the mental health issues in medical staff have been issued in China (Kang et al., 2020), some of the staff refused to receive mental health care (Chen et al., 2020).

It has been reported that the incidence of anxiety and depression was high among the medical staff during the outbreak of COVID-19, with prevalence rates of anxiety and depressive symptoms being 44.6% and 50.4%, respectively (Lai et al., 2020). Due to the stigma around mental health problems in China (Bai et al., 2004), many members of the medical staff community were reluctant to accept professional psychological help (Chen et al., 2020). However, they preferred to seek help from their family members. Some medical staff was far away from their families because of the fear of infection, and they were reluctant to have close contact with them. Others were isolated and cannot return home for a long time (Raven et al., 2018). It has shown that family support is very important for medical staff involved in epidemic prevention (Mohindra et al., 2020). However, there has been no research on how family members can help medical professionals suffering from mental health issues and what kind of family environment can be useful in relieving negative emotions in these individuals during an epidemic. The family, which is the basic social unit, may affect the mental health of family members (Cheng et al., 2017). In Chinese culture, family relations are highly valued, and family is a very important support system (Poulin et al., 2012). It means that the importance of family is the core feature for most Chinese people. Previous studies have shown that the family environment can directly affect the emotions in family members, especially the dimension of cohesion (Harris and Zakowski, 2003; Burnett et al., 2017), expressiveness (Luebbe and Bell, 2014), and conflict (Yap et al., 2014; Yap and Jorm, 2015; Fosco et al., 2016). However, whether the family environment has an effect on the mental health of medical staff during the epidemic is still not clear.

Self-efficacy refers to an individual’s judgment about his or her ability to complete a certain task successfully, and it reflects the self-confidence of an individual to cope with various difficulties and setbacks in life (Tang et al., 2019). Previous research has shown a positive correlation between the family environment and self-efficacy (Mahmoudi, 2012). Individuals with a better family environment were shown to be more likely to have higher levels of self-efficacy (Hemati et al., 2020). For example, individuals can freely communicate with family members, express more about themselves, and have a frequent high contact of intimacy with other family members, which will lead to a high level of self-efficacy (Hemati et al., 2020). Studies show that increasing self-efficacy is an essential aspect of the psychological intervention to protect the mental health of individuals (Caldwell et al., 2009). It seems that the higher the self-efficacy in an individual, the better the mental health (Sebastian, 2013; Jiang et al., 2020). High self-efficacy can improve an individual’s mental health (Bandura, 2012). However, a low level of self-efficacy harms a person’s psychological well-being (Cieslak et al., 2008; Sachs-Ericsson et al., 2011). Importantly, studies have shown that self-efficacy is one of the critical factors that modulate an individual’s emotions, especially feelings of anxiety and depression (Bandura et al., 1982; Kanfer and Zeiss, 1983; Cybulski et al., 2017; Schönfeld et al., 2019). These studies suggested that higher levels of self-efficacy are associated with lower levels of feelings of anxiety and depression. During the outbreak of SARS, medical staff with low self-efficacy often had a higher fear of the epidemic, which was positively correlated with their poor mental health status (Ho et al., 2005). Self-efficacy can predict the significant difference in mental health during the epidemic. The lower the self-efficacy, the worse the mental health status (Yıldırım and Güler, 2020). It has been found that lower psychological stress among dentists during the COVID-19 epidemic is associated with being in a stable relationship and having a higher sense of self-efficacy (Shacham et al., 2020). It suggests that family relationships and self-efficacy during the epidemic may both affect the mental health of medical staff. The partial mediating role of self-efficacy in some psychological trait relationships has been supported by relevant research (Haj-Yahia et al., 2019). Indeed, it has been found that self-efficacy was a mediator for the association of daily stress and mental health (Schönfeld et al., 2019). However, there has been no study focusing on the relationship between family environment, self-efficacy, and the emotional state of medical professionals during an epidemic. The relationship between family environment, self-efficacy, and depression and anxiety, and whether the influence of family environment on anxiety and depression is regulated by self-efficacy needs to be further studied.

Therefore, the purpose of this study was to further explore the direct and indirect impact of the family environment on symptoms of anxiety and depression among the medical staff involved in controlling the epidemic. When examining the indirect effects, we took self-efficacy as an intermediary variable. Mediating effect analysis was performed to explore the role of self-efficacy in the relationship between family environment and symptoms of anxiety and depression. Based on our findings, we provide viable strategies for the family based psychological intervention of the medical staff during an epidemic that will help to improve our psychological crisis intervention system.

## Materials and Methods

### Participants

This research was a cross-sectional study using the convenience sampling method to collect survey results through an online questionnaire. The online survey was conducted in Beijing from February 28, 2020 to March 9, 2020. Inclusion criteria for the study were as follows: (1) Chinese, working in Beijing; (2) 18–65 years old; (3) medical staff including doctor or nurse involved in COVID-19 epidemic prevention and control; and (3) has read and agreed to the online informed consent. The protocol of this study was approved by the Ethics Committee of the Beijing Huilongguan Hospital.

### Study Instruments

#### The Family Environment Scale-Chinese Version (FES-CV)

This scale was based on the Family Environment Scale (FES) developed by Moss (Moos and Moos, 1994), which was translated into Chinese by Wang et al. (1999). The scale has 90 entries in total, including 10 dimensions (cohesion, expressiveness, conflict, independence, achievement orientation, intellectual-cultural orientation, active-recreational orientation, moral-religious emphasis, organization, and control), with nine entries for each dimension. Each entry has a true and false option. The higher the score of a particular dimension, the more prominent the characteristics of the family in that aspect. The scores of conflict and control were negatively correlated with the family environment, whereas others positively correlated with the family environment. This scale has been shown to have good structural, content, and external validity when applied to the Chinese population (Phillips et al., 1998). In this study, we selected three dimensions of this scale that were closely related to emotions investigated in previous studies: cohesion, expressiveness, and conflict (Harris and Zakowski, 2003; Burnett et al., 2017).

#### The General Self-Efficacy Scale (GSES)

This scale was developed by German psychologist Schwarzer (1995), which was translated and revised for the Chinese version by Wang et al. (2001). The scale comprises of 10 items with four answer options, and the answer options range from 1 (not at all true) to 4 (completely true). Higher scores mean higher levels of self-efficacy; a score of 1.0–2.0 means low level, 2.1–3.0 means medium level, and 3.1–4.0 means a high level of self-efficacy. The revised scale has been shown to have excellent reliability and validity in the Chinese population (Wang et al., 2001). The Cronbach’s alpha of this scale is 0.87, the retest reliability is 0.83, and the correlation coefficient between the 10 items and the total scale score is 0.60–0.771.

#### The Generalized Anxiety Disorder Scale-7 (GAD-7)

This scale was developed by Spitzer et al. (2006). Previous research demonstrated that the Chinese version of GAD-7 has good reliability and validity, and the sensitivity and specificity of this version were 0.86 and 0.95, respectively (Huang et al., 2019). This scale is composed of seven items, and each item has a 0–3 points scale. The total score range is 0–21 points; 0–4 for no anxiety, 5–9 for mild anxiety, 10–14 for moderate anxiety, and more than 15 for severe anxiety.

#### The Patient Health Questionnaire-9 (PHQ-9)

The scale was developed based on the fourth edition of the Diagnostic and Statistical Manual of Mental Disorders (Kroenke et al., 2001). We used the Chinese version of this scale. This scale is composed of nine items, and each item has a 0–3 points scale. The symptom severity is determined by the total score, with 5–9 being mild, 10–14 being moderate, 15–19 being moderately severe, and 20–27 being severe. Cronbach’s alpha of the PHQ-9 in the Chinese population is 0.86, and the retest reliability is 0.86, which indicates that this test has excellent reliability and validity (Wang et al., 2014).

### Statistical Analysis

All of the analyses were performed using SPSS for Windows 23.0. We reported means and standard deviations for continuous variables showing normal distribution and frequencies and proportions for categorical variables. The original scores of the dimensions of conflict in FES-CV were not normally distributed and were presented as medians and quartiles. A chi-squared test was used to test the relationship between the demographic data of the participants and their anxiety and depressive symptoms. The study participants were grouped based on whether they showed/did not show anxiety or depressive symptoms. The inter-group comparison of the scores of FES-CV and GSES was carried out using the independent sample *t*-test and the independent sample Kruskal-Wallis test. Spearman correlations were calculated to determine the relationships between the scores of the various scales. We categorized the family environment (cohesion, expressiveness, and conflict) as an independent variable, anxiety and depression symptoms as dependent variables, and self-efficacy as an intermediary variable. After controlling for demographic variables, the direct, indirect, and total effects of the family environment on the symptoms of anxiety and depression were examined. The mediation analysis was run on the PROCESS macro for SPSS (Preacher and Hayes, 2004), using 5,000 bootstrap samples for bias correction and to establish 95% confidence intervals. All of the tests were two-tailed, and the significance level was set at *p* < 0.05.

## Results

The medical staff involved in this study mainly includes two groups. One is the staff of the hospital involved in treating patients infected with COVID-19; the other is the staff at the isolation point, whose main task is nucleic acid testing and medical services for the quarantined personnel. A total of 653 medical staff members completed the online questionnaire, out of which six individuals did not complete the basic information, and two took more than 5 min to answer the questionnaire. Thus, 645 medical staff members participated in the study, of which 485 (75%) were women, and 160 (25%) were men, aged 21–65, with an average age of 35.88 ± 8.64. Most of the participants were under 40 (75%), had a bachelor’s degree (61%), were married (73%), and were living with their families (83%). Among these participants, 251 (39%) had anxiety symptoms, and 215 (33%) had depressive symptoms. The symptoms of anxiety and depression were closely related to the gender of the medical staff and whether they lived with their family members or not. The prevalence rate of anxiety and depression symptoms was higher in women (*X^2^* = 9.25, *p* = 0.002; *X^2^* = 4.09, *p* = 0.043) and in those who did not live with their families (*X^2^* = 5.38, *p* = 0.02; *X^2^* = 4.35, *p* = 0.037) (Table 1).

**TABLE 1:** Demographic characteristics of the study participants (*N* = 645).

**Variables**	**Total**	**Anxiety symptoms**		**Depressive symptoms**	
	***N* = 645**	***X*^2^**	***P***	***X*^2^**	***P***
Gender		9.25	0.002	4.09	0.043
Male	160				
Female	485				
Age		6.30	0.098	5.97	0.113
≤30	208				
31–40	274				
41–50	119				
≥51	44				
Education		2.71	0.607	2.39	0.664
Below bachelor’s	126				
Bachelor’s	396				
Master’s or higher	123				
Marital status		1.55	0.213	0.96	0.326
Single	176				
Married	496				
Live with family		5.38	0.02	4.35	0.037
Yes	535				
No	110				

We divided the participants into two groups: one group with anxiety symptoms (GAD-7 score > 4) and the other group without anxiety symptoms (GAD-7 score ≤ 4). We then investigated the significance of the differences in the scores of the two groups for the FES-CV and GSES scales. Next, the participants were divided into two groups according to whether they had depressive symptoms or not (depressive symptoms: PHQ-9 score > 4; no depressive symptoms: PHQ-9 score ≤ 4). Like the anxiety symptoms, we investigated the significance of the difference between the scores of FES-CV and GSES scales in the two groups. The results, which show the significant differences among groups, are shown in Table 2.

**TABLE 2:** Differences in scores of the family environment and self-efficacy under different emotions (*N* = 645).

**Variables**	**Anxiety symptoms**			**Depressive symptoms**		
	**Yes**	**No**		**Yes**	**No**	
				
	**M (SD)/M (P25, P75)**		***t/Z***	**M (SD)/M (P25, P75)**		***t/Z***
**FES-CV**						
Cohesion	7.05 (2.25)	8.12 (1.35)	6.76***	6.64 (2.31)	8.20 (1.28)	9.09***
Expressiveness	5.34 (1.79)	5.97 (1.49)	4.66***	4.90 (1.78)	6.11 (1.42)	8.57***
Conflict	2 (1.3)	3 (2.5)	–6.12***	2 (1.3)	3 (2.5)	–6.12***
GSES	2.53 (0.56)	2.76 (0.55)	5.15***	2.47 (0.54)	2.76 (0.55)	6.26***

***P < 0.001. FES-CV, The Family Environment Scale-Chinese Version; GSES, The General Self-Efficacy Scale.

Next, we used correlation analysis to determine the correlation between the scores of each scale. The results showed that there were statistically significant relationships between the anxiety and depressive symptoms of medical staff, their family environment, and their sense of self-efficacy. Anxiety and depressive symptoms showed a significant positive correlation with the dimension of conflict in FES-CV scale (*r* = 0.29, *p* < 0.001; *r* = 0.25, *p* < 0.001), and a significant negative correlation with the dimension of cohesion (*r* = −0.31, *p* < 0.001; *r* = −0.38, *p* < 0.001), expressiveness (*r* = −0.23, *p* < 0.001; *r* = −0.30, *p* < 0.001), and self-efficacy (*r* = −0.25, *p* < 0.001). More specifically, individuals with bad family environments and low self-efficacy were more likely to show symptoms of anxiety and depression. Besides, self-efficacy positively correlated with the dimension of cohesion and expressiveness and negatively associated with the dimension of conflict (Table 3).

**TABLE 3:** Correlations between the factors of different scales (*N* = 645).

**Variables**	**Cohesion**	**Expressiveness**	**Conflict**	**General self-efficacy**	**Anxiety symptoms**	**Depressive symptoms**
Cohesion	-					
Expressiveness	0.44***	–				
Conflict	–0.32***	–0.14**	–			
General self-efficacy	0.24***	0.15***	–0.11**	–		
Anxiety symptoms	–0.31***	–0.23***	0.29***	–0.25***	–	
Depressive symptoms	–0.38***	–0.30***	0.25***	–0.25***	0.72***	–

**P < 0.01; ***P < 0.001.

**FIGURE 1:**
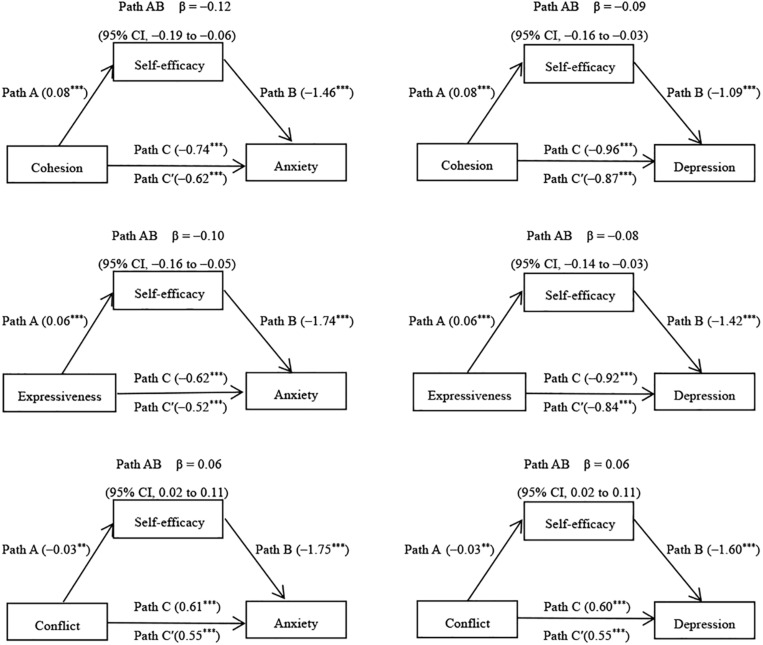
Mediation effects of self-efficacy in the relationship between family environment and the symptoms of anxiety and depression (*N* = 645). **P < 0.01; ***P < 0.001.

After controlling for demographic variables, we examined the mediating effects of self-efficacy (Figure 1). Self-efficacy was significantly associated with the symptoms of anxiety and depression. It significantly mediated the association between the family environment and anxiety symptoms (β = −0.12; 95% CI, −0.19 to −0.06; β = −0.10; 95% CI, −0.16 to −0.05; and β = 0.06; 95% CI, 0.02 to 0.11). Similarly, it mediated the association between family environment and the depressive symptoms (β = −0.09; 95% CI, −0.16 to −0.03; β = −0.08; 95% CI, −0.14 to −0.03; and β = 0.06; 95% CI, 0.02 to 0.11). When controlling for self-efficacy, the association between the family environment and anxiety symptoms were still significant (β = −0.62, *p* < 0.001; β = −0.52, *p* < 0.001; and β = 0.55, *p* < 0.001) and similarly for family environment and depressive symptoms (β = −0.87, *p* < 0.001; β = −0.84, *p* < 0.001; β = 0.55, *p* < 0.001). Thus, self-efficacy partly mediated the relationship between the family environment and the symptoms of anxiety and depression.

## Discussion

In the current study, we conducted an online questionnaire survey of some medical staff involved in COVID-19 prevention and control in hospitals and isolation sites in Beijing. We found that a considerable proportion of medical professionals had anxiety (39%) and depressive symptoms (33%), as noted in previous studies (Huang et al., 2020; Lai et al., 2020). Moreover, our results showed that the family environment of medical staff and their symptoms of anxiety and depression during the epidemic were closely related to self-efficacy, and self-efficacy partly mediated the relationship between the family environment and the symptoms of anxiety and depression.

In this present study, we found that the self-efficacy of male medical staff was significantly higher than that of female medical staff (*t* = 3.245, *p* = 0.001). However, the symptoms of anxiety (*p* < 0.001) and depression (*p* = 0.002) during COVID-19 were significantly lower than that of female medical staff, which was consistent with previous research results. For example, it showed that female medical workers experience higher levels of anxiety, depression, and distress during COVID-19 (Lai et al., 2020). It also reported that the self-efficacy of male medical staff is significantly higher than that of female medical staff (Tang et al., 2019). It should also be noted that medical staff who did not live with their families were more likely to have symptoms of anxiety and depression during the epidemic. Thus, our study suggests that we should pay more attention to such medical staff and provide them psychological intervention. This observation also indicates that the family plays a certain role in regulating negative emotions. Furthermore, our study showed a close relationship between the family environment of the medical staff and their symptoms of anxiety and depression. The medical staff members with low cohesion and expressiveness, as well as high conflict in the family environment, were more likely to show anxiety and depressive symptoms during the epidemic.

Notably, in this present study, the relationship between the family environment and the symptoms of anxiety and depression reveals that the family environment can, directly and indirectly, affect the emotions of medical staff during an epidemic. The family environment can significantly predict the emergence of anxiety and depressive symptoms directly, which is consistent with previous results. For example, some studies found that there was a correlation between cohesion in the family environment and depression in family members (Burnett et al., 2017); families with high cohesion, which have high levels of family support and ties, likely reduce depression (Park et al., 2018; Cano et al., 2020). In contrast, low family cohesion and conflict between parents increased the risk of depression and anxiety in family members (Park et al., 2018; Cano et al., 2020). In families with a high degree of cohesion, individuals can get more psychological help and emotional support within the family (Birgisdóttir et al., 2019), so that the psychological pressure can be appropriately relieved. Positive emotional expression within the family can prevent suppression of inner feelings and buffer internal conflicts, especially in the face of stressful events. In contrast, negative emotional expression and low emotional expression within the family are associated with higher anxiety and depression (Luebbe and Bell, 2014; Park et al., 2018). In a high-conflict family, family members are prone to conflict between each other, leading to anxiety. Therefore, the results in this study support the hypothesis that the family environment can influence the emotional state of the family members and that a negative family environment is a psychological risk factor for the rising emotional distress of the medical staff during an epidemic.

Additionally, the influence of the medical staff’s family environment on their symptoms of anxiety and depression during the COVID-19 epidemic is partly through the role of self-efficacy, which means self-efficacy plays a critical role in mediating the effect of family environment on symptoms of anxiety and depression. Indeed, previous studies supported that self-efficacy had a protective effect on mental health (Bandura, 2012) and played a vital role in the regulation of stress (Bandura et al., 2003). High self-efficacy was related to better psychological adjustment (Bandura, 2012) and lower emotional distress (Benight and Harper, 2002). Individuals with high self-efficacy had positive expectations and beliefs, had successful experiences, generated positive emotions, and were more likely to seek psychological support to modulate their emotions when facing stressful situations (Tsang et al., 2012). A bad family environment can reduce an individual’s self-confidence and ability (Hemati et al., 2020). Self-efficacy is the embodiment of such confidence and ability (Tang et al., 2019). That is to say, the family environment affects self-efficacy by affecting people’s self-confidence and ability, thus affecting individuals’ behavioral patterns and emotional responses to stress (Tsang et al., 2012). For example, a medical worker with a good family environment has confidence in the success of the fight against the epidemic and also believes that he is capable of doing his job, which will ease his fear of the epidemic and anxiety about the high-risk work of infection. Self-efficacy played a partial mediating role between the family environment and symptoms of anxiety and depression, indicating the existence of other variables between them. Future studies should, therefore, include other relevant variables that are likely involved in the relationship between the family environment and negative emotions. This study indicates that adjusting self-efficacy is a meaningful way to regulate the anxiety and depressive symptoms of medical staff during an epidemic.

Because of the close correlation between the family environment and the symptoms of anxiety and depression of medical staff during an epidemic, we need to pay more attention to psychological assistance for medical staff from the perspective of their family situation. When providing psychological assistance to medical staff during the epidemic, we should not only focus on the medical staff but also care about their family members and family relations. By improving the family environment and increasing the active support of the family, their emotional problems can be effectively alleviated (Mohindra et al., 2020). The focus of the medical staff’s treatment of family relations should be to enhance the intimacy between family members, increase their interaction, encourage them to talk to each other, resolve the family conflicts in time, and create a good family atmosphere. Based on the results of this study, we propose the following suggestions for medical staff. First, we suggest that medical staff should have time to communicate with their families and that they should be encouraged to share their feelings with family members and get their support and encouragement. For example, they should be encouraged to record their routines in the hospital and share them with their families (Chen et al., 2020). The hospital or isolation point shall provide relevant communication conditions and equipment for this purpose. Second, during the epidemic period, the staff of the relevant departments of the hospital should be aware of the difficulties existing in the family of medical staff, and they should guide these staff members and help them solve those problems to avoid family conflicts. Third, the family members of medical staff should be aware of the mental health issues of the staff member. Family safety plays the most important role in reducing the pressure of medical staff during the epidemic (Cai et al., 2020). Therefore, the staff members should stay connected with their families through WeChat, SMS, and other apps to understand their health status, which will help lessen the negative mental state of the medical staff during the COVID-19 pandemic outbreak. These Suggestions can bring medical staff closer to their families, have more emotional communication, and reduce family conflicts. With the implementation of these measures, the medical staff’s sense of self-efficacy will also be improved.

Besides, our results suggest that improving self-efficacy will help to alleviate the anxiety and depressive symptoms of medical staff during the COVID-19 outbreak. Manipulating self-efficacy is an important way to prevent mental health problems when dealing with stress (Schönfeld et al., 2019). Previous studies have focused on the effects of self-efficacy on the mental health and work quality of medical staff (Amiri et al., 2019; Tang et al., 2019), and it suggested that necessary interventions should be implemented to improve the self-efficacy of medical staff. In the prevention and control of COVID-19, medical staff is faced with two main difficulties. On the one hand, medical staff has heavy work tasks, great pressure, high risk of infection, and lack of support (Spoorthy et al., 2020). On the other hand, most of the medical staff are required to be isolated in hospitals or isolation points. Their families will face more prominent problems (Mohindra et al., 2020). Some positive motivation factors can boost morale and improve the self-efficacy of medical staff, such as family and social support, positive example, recognition, and appreciation from others, successful experience, self-identity (Spoorthy et al., 2020). Positive feedback and encouragement from others could also effectively improve self-efficacy (Bandura, 1997; Zinken et al., 2008; Brown et al., 2012). The pre-job training, encouragement from colleagues and family, affirmation from patients and society, and sufficient material support were all helpful ways to improve the self-efficacy of medical staff during the COVID-19 outbreak. Medical staff in a good family environment can get better family support. The support reduces the sense of uneasiness caused by isolation, and improve self-efficacy, increase work confidence, improve work efficiency and quality, and reduce the negative emotions caused by epidemic infection.

It has been reported that the mental health status of Chinese medical staff is poor (Zhou et al., 2018), and they are exposed to immense workplace pressure and face complex doctor-patient relationships. The reason lies in the contradictions in the current medical system reform in China, such as the uneven distribution of medical resources (Lu et al., 2019), the disequilibrium between health care needs and medical development (Zhou et al., 2018), and the imperfection of the medical system (Ta et al., 2020). During an epidemic period, protecting the mental health of the medical staff would benefit their health as well as the control of the epidemic worldwide (Kang et al., 2020). The National Health Commission of China has published a national guideline of psychological crisis intervention for COVID-19, which is guided for the protection of the mental health of the medical staff (Kang et al., 2020). However, the family environment is particularly important to the mental health of the medical staff, and self-efficacy plays an important role in regulating the relationship between them. Appropriate guidelines should be issued nationally to improve the family environment of the medical staff and for the improvement of their self-efficacy.

There are some limitations to the current study that need to be addressed. First, there are limitations to the method of sampling. Sampling bias may have occurred by using a convenient sampling method. Second, we have a small sample size, and all participants are from Beijing, so the research participants in this study may not be sufficiently representative of the population we are interested in studying, which may limit the conclusion of research results. Third, online questionnaire surveys cannot observe the participants’ answering process, there is the possibility of random answer and perfunctory answer, cannot guarantee the complete authenticity of data. Fourth, we did not measure other potential confounding variables that may exist between the family environment and the emotional state of medical staff during the COVID-19 outbreak. Finally, the researchers are all medical staff, and the design of the survey may be more based on clinical observation. In the future, the research design can be combined with clinical observation and the existing theoretical framework.

## Conclusion

In the current study, we found that the anxiety and depressive symptoms of medical staff during the COVID-19 outbreak was closely related to their family environment, and their self-efficacy regulated the relationship between them. This study provides a new direction for the psychological intervention in medical staff during the epidemic that mainly focuses on improving their family environment and their self-efficacy.

## Data Availability Statement

The raw data supporting the conclusions of this article will be made available by the authors, without undue reservation, to any qualified researcher.

## Author Contributions

NH contributed to the manuscript writing. J-XC, YL, and S-SH contributed to the conception and designed the work. YL, L-LW, Y-YW, and LY contributed to the critical revision of the article. All authors contributed to the article and approved the submitted version.

## Conflict of Interest

The authors declare that the research was conducted in the absence of any commercial or financial relationships that could be construed as a potential conflict of interest.
